# Enhancing the Cherenkov over scintillation ratio using dichroic filters in BGO and TlCl for TOF-PET

**DOI:** 10.1038/s41598-025-01396-2

**Published:** 2025-05-28

**Authors:** Baharak Mehrdel, Nicolaus Kratochwil, Youngho Seo, Jarek Glodo, Pijush Bhattacharya, Gerard Ariño-Estrada, Javier Caravaca

**Affiliations:** 1 Department of Radiology and Biomedical imaging, University of California San Francisco, San Francisco, CA 94107 USA; 2 Department of Biomedical Engineering, University of California at Davis, Davis, CA 95616 USA; 3 Radiation Monitoring Devices, Inc, Watertown, MA 02472 USA; 4 Institut de Física d’Altes Energies - Barcelona Institut of Science and Technology, Bellaterra, Barcelona, Spain; 5 Lawrence Berkeley National Laboratory, Berkeley, CA USA

**Keywords:** BGO, TlCl, Cherenkov photons, Dichroic filters, Time of flight positron emission tomography, Characterization and analytical techniques, Imaging techniques, Imaging techniques

## Abstract

The Cherenkov emission in inorganic crystal scintillators has been shown to dramatically improve time resolution for time-of-flight positron emission tomography (TOF-PET) for in slow scintillators with a high refractive index such as bismuth germanium oxide (BGO). This is due to the faster nature of the Cherenkov emission (tens of picoseconds) with respect to scintillation (nanosecond). However, the presence of slower scintillation light and the inability of existing detectors to distinguish between Cherenkov and scintillation make it difficult for BGO to achieve a good CTR for all the detected coincidence events. In this paper, we exploit the difference between the Cherenkov and scintillation emission spectra and use dichroic filters to enhance the Cherenkov over scintillation ratio. Dichroic filters transmit or reflect photons based on their wavelength, with a photon attenuation lower than 10%. We investigate several shortpass and longpass dichroic filters in a single-photon configuration with BGO and find the optimal filter that maximizes the Cherenkov over scintillation ratio. We demonstrate that we can enhance the ratio of Cherenkov to scintillation photons by a factor of 2.17 ± 0.38 by employing a shortpass dichroic filter with a cut-off wavelength of 450 nm for BGO, and by a factor of 2.87 ± 0.40 using a longpass dichroic filter with a cut-on wavelength of 550 nm for iodine/beryllium-doped thallium chloride.

**Supplementary Information:**

The online version contains supplementary material available at 10.1038/s41598-025-01396-2.

## Introduction

Positron emission tomography (PET) is a commonly used non-invasive nuclear imaging modality. It is well known for its ability to provide functional interrogation for a number of conditions including cancer, neurological, and cardiovascular disorders^1–3^. The image quality of PET can be enhanced by introducing time of flight information (TOF-PET)^4–6^, which requires detectors with good time resolution at the sub-nanosecond level. Due to the capital importance of time resolution and energy resolution, development of fast and bright scintillators is an important topic for TOF-PET. In the last decades, Cherenkov radiation has been exploited in scintillators with slow decay times, such as bismuth germanium oxide (BGO), to enable their utilization in TOF-PET^7–13^. Materials with high indices of refraction are preferred in order to maximize the yield of Cherenkov photons. BGO, with a refractive index of ~ 2.1 at 450 nm and a transparency down to 310 nm, is a good candidate with a high Cherenkov yield and high gamma detection efficiency^14^. Another promising material is thallium chloride (TlCl) whose index of refraction is ~ 2.6 at 450 nm. The cut-off wavelength of TlCl is 380 nm which allows for transmission of Cherenkov photons in spectral band where the photon detection efficiency (PDE) is greater than 50% (peak sensitivity wavelength at 450 nm). When doped with beryllium (Be) and/or iodine (I), TlCl provides a scintillation component with emission spectra and yield that depends on the dopants and their concentration, as shown in [17]. This makes TlCl a promising scintillator for TOF-PET^15–17^ and proton range verification in proton therapy^18^.

An important challenge for the usage of Cherenkov light in scintillators is related to the fact that the number of emitted Cherenkov photons is several orders of magnitude smaller than that of scintillation photons^10,19,20^. Hence, Cherenkov photons are frequently undetected leading to the presence of long tails in the coincidence time measurements^12,20–22^. These tails can be mitigated by classifying fast and slow coincidences using the signal rise time in SiPMs^23^, but a significant slow component still remains.

**Fig. 1:**
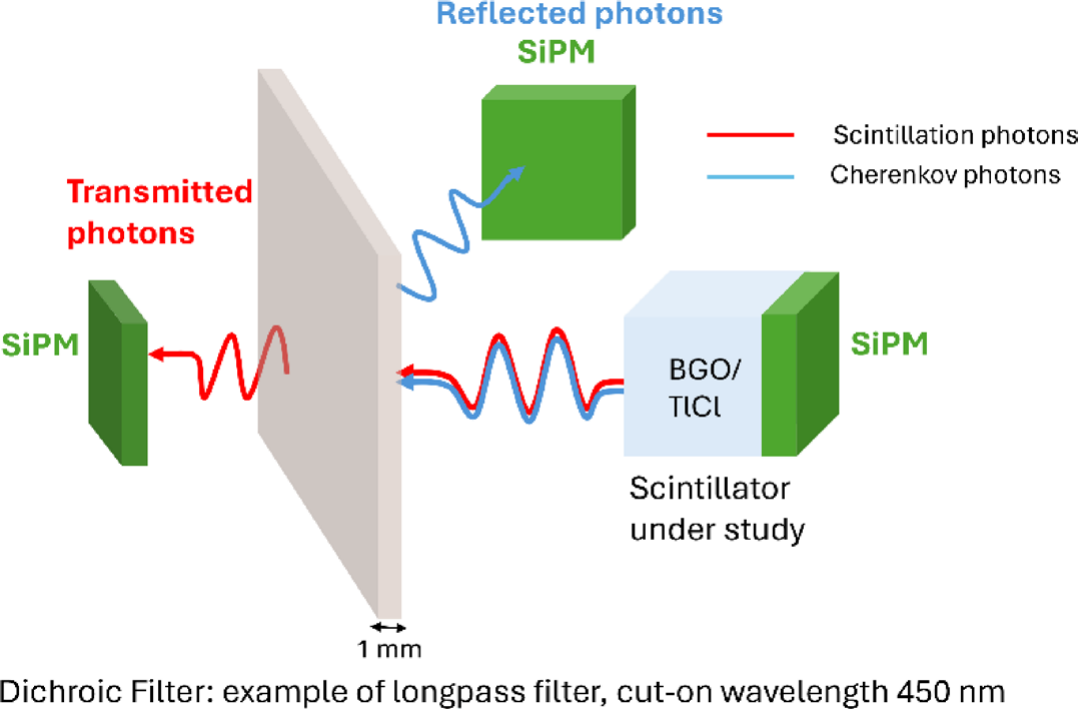
Sketch of potential utilization of dichroic filters to enhance the Cherenkov over scintillation ratio avoiding substantial attenuation of scintillation photons in the filter.

We propose to exploit the difference in the emission spectra between Cherenkov (faster) and scintillation (slower) in order to enhance the detected Cherenkov component over scintillation by using dichroic filters, inspired by similar efforts in high-energy physics^24–27^. Dichroic filters have the ability to transmit or reflect photons depending on their wavelengths, with an absolute photon attenuation value typically below 10%^28^. Hence, they could potentially be utilized to enhance the Cherenkov over the scintillation component to achieve better time resolution, as shown in the illustration of Fig. 1. To this end, regular optical filters have been proposed to select either the Cherenkov or the scintillation component produced in bismuth silicon oxide (BSO)^27^, but the attenuation of regular filters results in the part of the spectra not selected being lost, preventing for a concept like Fig. 1. Previous studies have also implemented dichroic mirrors for wavelength separation in laparoscopic multimodal imaging^29^ and regular bandpass filters for depth of interaction encoding in TOF-PET detectors^30^.

This work corresponds to the first step towards the concept in Fig. 1. We focus on the basic characterization of the different shortpass and longpass dichroic filters with BGO and I/Be-doped TlCl. We use a single-photoelectron configuration in order to measure the detected time distribution for each filter. From this, we calculate the relative photon detection efficiency, and the individual Cherenkov (fast) and scintillation (slow) components as a function of the cut-on (cut-off) shortpass (longpass) dichroic filters. We do this in two independent configurations, namely, a transmission and a reflection configuration, each corresponding to the two different paths in Fig. 1. Finally, we evaluate the enhancement of the Cherenkov over the scintillation in each case and compare it to the case without a filter.

## Materials and methods

### Scintillation crystals

**Fig. 2:**
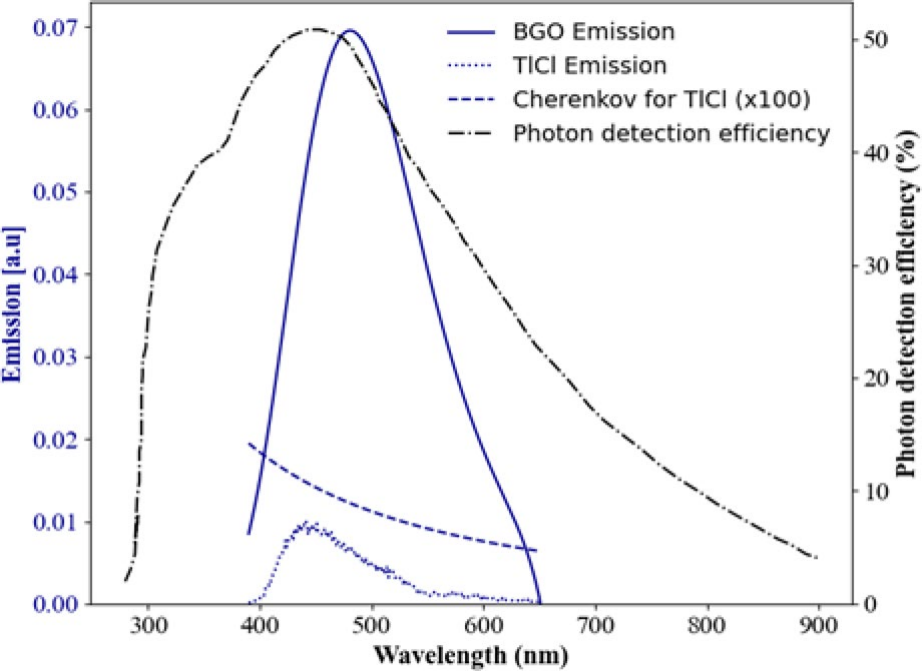
BGO, TlCl, and Cherenkov emissions for TlCl were reproduced from^14,17^. Photon detection efficiency was reproduced based on the SiPM datasheet.

We employ BGO and TlCl manufactured by Epic Crystal (China) and Radiation Monitoring Devices (Watertown, MA, USA), respectively. The crystal dimensions were 3 × 3 × 3 $\:{\text{m}\text{m}}^{3}\:$for BGO and 3 × 3 × 5 $\:{\text{m}\text{m}}^{3}$ for TlCl, with all faces polished. The scintillation emission spectra of BGO, TlCl and Cherenkov emission ($\:1/{\lambda\:}^{2}$) are displayed in Fig. 2. A lutetium fine silicate (LFS) scintillator crystal, obtained from ZECOTEK (Canada), was used as a time reference detector. Its dimensions were 3 × 3 × 20 $\:{\text{m}\text{m}}^{3}$ and all faces polished. Table 1 provides a summary of the relevant physical properties of the crystals used in our experiments.

**Table 1:** Relevant properties of scintillator crystals.

Crystals Properties	BGO	TlCl	LFS
Density [g/$\:{cm}^{3}$]	7.1	7.0	7.35
$\:{Z}_{eff}$	73	76	64
Refractive index @ 450 nm	2.1	2.6	1.81
^*^Cutoff wavelength (nm)	310	380	400
Peak emission @ Scintillation max (nm)	480	450	425
Scintillation light yield [photons/keV]	8.5	0.9 ± 0.3	80–85
Attenuation length of 511 keV photons (cm)	1.1	1.04	1.15
References	^31^	^17^	^32^

*Cutoff wavelength for scintillators refers to the shortest wavelength that the scintillator can effectively transmit. The photons with wavelengths shorter than the cutoff wavelength are not efficiently transmitted by the scintillator material.

### Dichroic filters

**Fig. 3:**
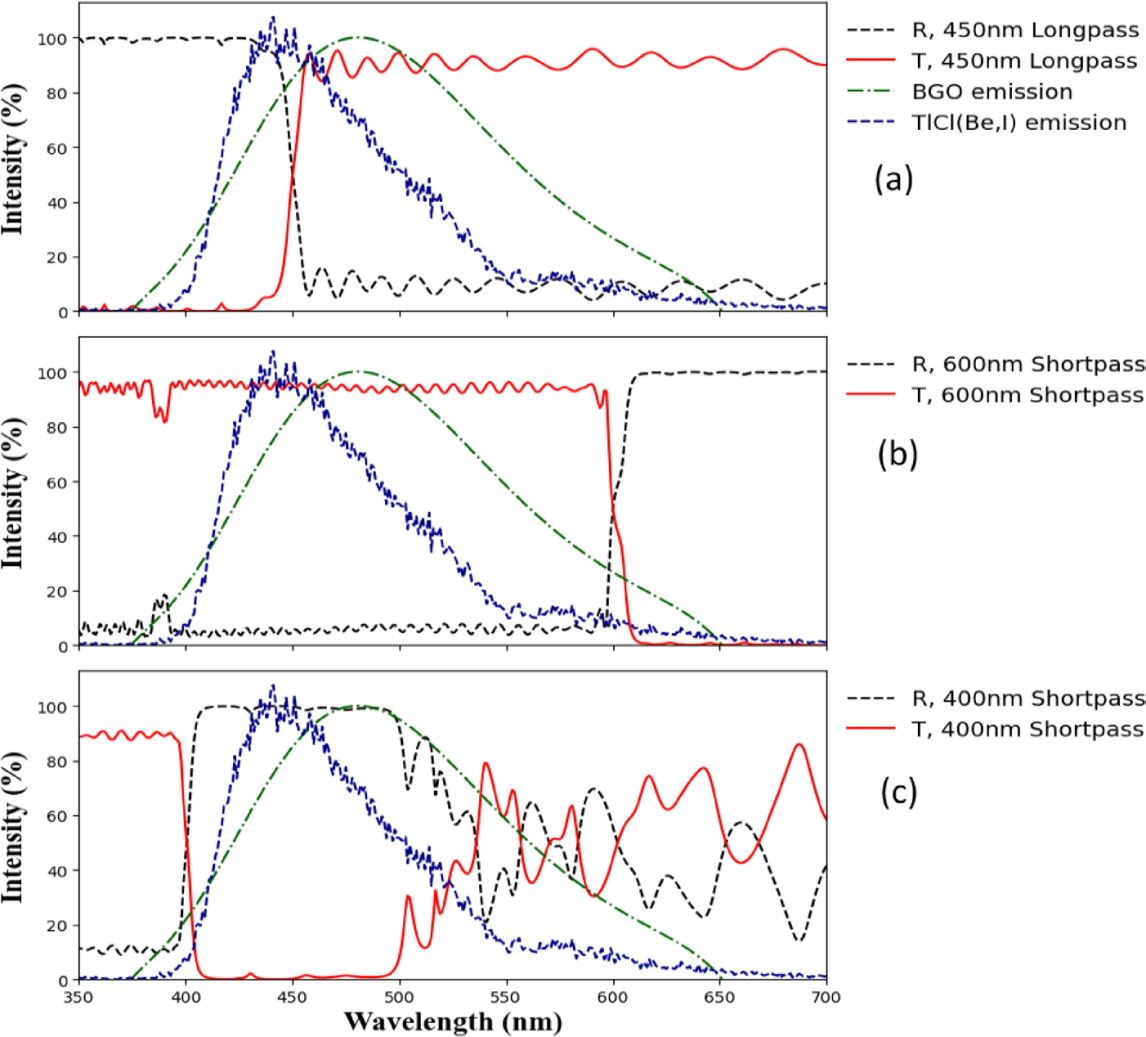
Shows the transmission (T, red solid line) and reflection (R, dashed line) curves of dichroic filters, provided with permission from Edmund Optics Inc. (**a**) a longpass dichroic filter has a cut-on wavelength of 450 nm. (**b**) a shortpass dichroic filter with a cut-off wavelength of 600 nm. (**c**) the 400 nm shortpass dichroic filter exhibits a wavelength-dependent oscillations in both transmission and reflection beyond 500 nm. (Green dash dot refers to BGO emission and blue dash line refers to TlCl (Be, I) emission).

In this research work, two types of dichroic filters were employed. Longpass dichroic filters allow about 90% of the light being transmitted above a specific cut-on wavelength, while reflecting 100% of shorter wavelength (Fig. 3a). Conversely, shortpass dichroic filters permit the transmission of 90% light below a specific cut-off wavelength while reflecting 100% of longer wavelengths (see Fig. 3b). As evident from Fig. 3c, the 400 nm shortpass dichroic filter exhibits a wavelength-dependent oscillations in both transmission and reflection beyond 500 nm. Table 2 provides detailed information on each filter, including its manufacturer and size.

**Table 2:** Dichroic filters properties.

Dichroic Filters							
Shortpass				**Longpass**			
Cut-off wavelength (nm)	Manufacture	Dimensions (mm)	Model number	Cut-on wavelength (nm)	Manufacture	Dimensions (mm)	Model number
400	Ed^1^	25.2 × 35.6	69,212	409	Ed	25.2 × 35.6	86,330
414	KO^2^	50 × 50	414 FDS50	420	KO	50 × 50	420 FDS50
450	KO	50 × 50	450 FDS50	450	Ed	25.2 × 35.6	69,898
500	KO	50 × 50	500 FDS50	500	KO	50 × 50	500 FDL50
550	KO	50 × 50	550 FDS50	550	KO	50 × 50	550 FDL50
600	Ed	25.2 × 35.6	69,216	600	KO	50 × 50	600 FDL50

^1^Ed: Edmund Optics (https://www.edmundoptics.com/c/dichroic-filters/1344/).

^2^KO: Knight Optical (https://www.knightoptical.com/stock/default/filters/dichroic-filters.html).

### Coincidence acquisition setup

**Fig. 4:**
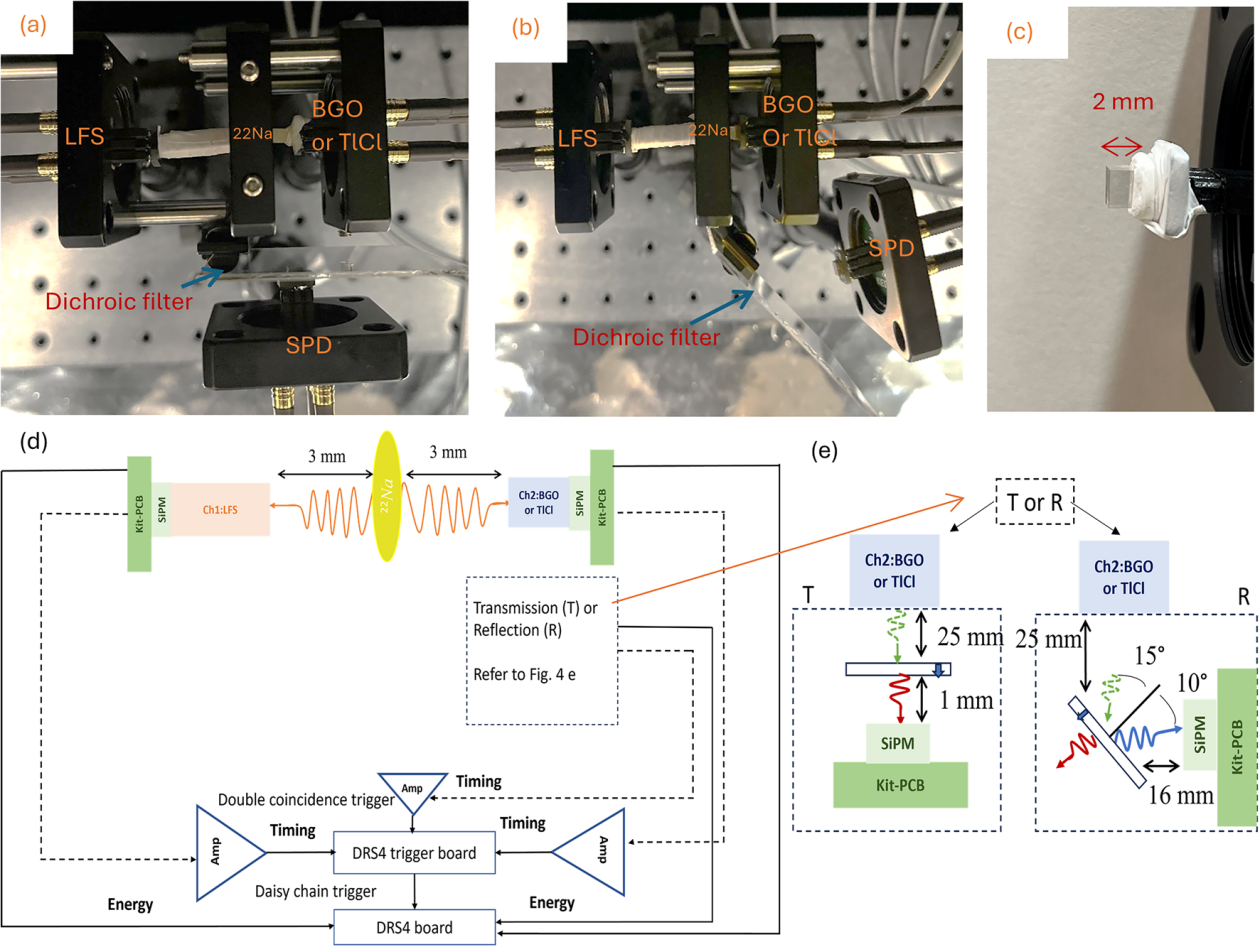
Photographs of the transmission (**a**) and reflection (**b**) configurations. Photo of scintillator crystal under the test was partially wrapped in Teflon (**c**). Schematic of the configuration and data acquisition setup (**d**). The transmission and reflection setup with the distance and angle of incident light, green dotted line refers to incident light, the red line refers to transmitted light, and the blue line refers to reflected light (**e**).

A coincidence setup consisting of a time reference crystal (LFS) and the crystals under study (BGO or TlCl) was assembled to characterize the time spectra of detected photons in the presence of dichroic filters. Each scintillator crystal was optically coupled to the 3 mm × 3 mm face to a single SiPM (S14160-3050HS, Hamamatsu, Japan) using Meltmount, which has a refractive index (n) of 1.58 ^27^. The SiPMs employed in this setup are instrumented at a 2 V over-voltage. We chose this low over-voltage to have a relatively high gain while reducing the dark noise component, which is crucial in our single-photon experiments. However optimal for our purpose, this low over-voltage negatively impacts the SiPM time resolution, so a higher over-voltage should be used in future multi-photon measurements when characterizing the time resolution. A third SiPM intended for a single-photon detection (SPD) analysis is located at a distance from the BGO or TlCl crystals. To maximize the light collection and provide a time reference, the LFS was wrapped with more than 5 layers of Teflon. The BGO and TlCl crystals were partially wrapped in Teflon at the interface between the SiPM and the crystal (around 1 mm) in order to allow photons to escape the crystal towards the SPD (Fig. 4c).

The LFS time reference crystal and the BGO or TlCl crystal were positioned on each side of a 10 µCi^22^Na source, allowing back-to-back 511 keV gamma-ray emissions to be detected in coincidence (Fig. 4a, and b). Our studies were performed in two different configurations inside a light-tight dark box: transmission and reflection. For the transmission measurements, dichroic filters were placed between the BGO or TlCl crystal and the SPD (Fig. 4e). In this setup, the distance between the dichroic filter and SPD was 1 mm. For the reflection measurements, the filter was rotated 45 degrees respect to the first position (transmission configuration), the SPD located in front of dichroic filter at distance of 16 mm away from filter (see Fig. 4b and e). The exact angles between the incident light and reflected light are presented in Fig. 4e. Half a million of coincidence triggers were acquired per configuration, filter and scintillator combination. The acquisition time was approximately 27 h and coincidence rate were varied depending on specific configuration, and scintillators under test but averaged around 5 counts per second. The SiPMs were biased by a DT5485P voltage power supply (CAEN, Italy) to 40 V for both BGO and TlCl experiment to balance noise and gain. Signals from SiPMs were read out using a commercial silicon photomultiplier evaluation kit (PEVAL-KIT-MCX) PCB (Ketek, Munich, Germany). The signal was split into two branches: 90% of the signal used for timing measurement branch and 10% for energy measurement branch, allowing for simultaneously acquisition of timing and energy. Timing signals were connected to the low noise preamplifier (ZFL-1000LN+) from Mini Circuits (USA). DRS4 evaluation boards V5 (designed at the Paul Scherrer Institute, Switzerland) digitized three signals, with the master trigger DRS4 set to trigger on the coincidence between the timing channels of the reference detector LFS and the BGO or TlCl crystals^33,34^ (Schematic of acquisition setup Fig. 4d). Full waveforms were digitized for offline signal processing to obtain energy via integration and time via leading edge threshold.

A measurement of dark noise was carried out by covering the SPD with a dark opaque cloth. Time distribution of dark noise was calculated by employing a timing and event selection (see section coincidence event selection section).

### Waveform analysis

#### 1) digitization

**Fig. 5:**
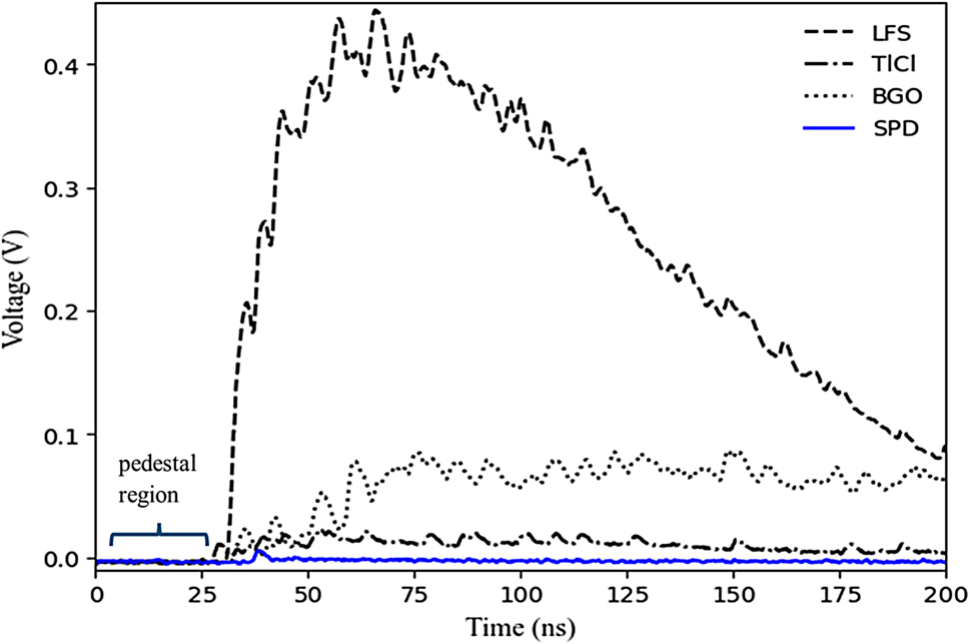
Examples of a single waveform for the LFS time reference detector (dashed line), the SiPM attached to BGO (dotted line) and TlCl (dash-dotted line), and the single photon SiPM (blue solid line).

The waveforms were digitized with the DRS4 evaluation boards at a sampling rate of 5 GS⁄s and with 1024 sampling points, which allowed us to collect 200 ns waveforms. We set the trigger logic in the master DRS4 to trigger off of the leading-edge of the coincidence signal between the SiPM coupled to the LFS and the one coupled to the BGO or TlCl crystal. We used a trigger threshold of 0.4 V for the LFS reference detector and of 0.05 V and 0.005 V for BGO and TlCl, respectively. The SiPM single photoelectron average signal corresponds to ~ 7 mV at 40 V SiPM bias voltage. Due to the much lower light yield of TlCl, the threshold was set below the single photoelectron signal. Waveform examples for detected coincidence events are shown in Fig. 5. It is important to note that this configuration does not involve triggering on SPD, which normally requires a considerably lower threshold.

#### 2) charge integration

For charge integration, only waveforms that exhibited baseline fluctuations below 5 mV in the pedestal region (Fig. 5) were selected. Subsequently, the charge was calculated as the pedestal-corrected integral of the waveform signal (w(t)). The pedestal, P, for each waveform was computed according to Eq. (1).

1 $$\:P=\:\frac{\sum\:_{{i=\:P}_{start}}^{{P}_{end}}{w\left(t\right)}_{i}}{{P}_{end}-{P}_{start}}$$

where $\:{P}_{start}$ and $\:{P}_{end}\:$are the initial and the last samples, corresponding to 1 to 61 (12 ns) for BGO and 1 to 46 (9 ns) for TlCl. The integrated charge, Q, is calculated as

2 $$\:Q=\:{\sum\:}_{i={int}_{start}}^{{int}_{end}}{w\left(t\right)}_{i}-P\times\:\left({int}_{end}-{int}_{start}\right)$$

where the charge integration region was defined by $\:{int}_{start}$ and $\:{int}_{end}$ and corresponded to 62 to 1024 (192 ns) for BGO and 47 to 1024 (195 ns) for TlCl. Different $\:{int}_{start}$ values are chosen to accommodate the different scintillation rise and decay times of each scintillator. Based on the literature, the rise and decay times of TlCl^34^ are shorter than BGO^16,35,36^, which justifies the later $\:{int}_{start}$ of 62 samples.

#### 3) timing

A time at fixed threshold is used for both SPD and other SiPMs. Initially, the waveform is pedestal-corrected by employing Eq. (1) in order to minimize baseline fluctuations that could affect the threshold detection. Then, the algorithm identifies the maximum sample in the waveform and searches backward to find the last sample below a 0.005 V threshold, which roughly corresponds to 50% of a single photon signal. The time is then interpolated between the two samples surrounding the threshold crossing employing linear interpolation algorithm^37^. This linear interpolation enhances the precision of the timing calculation, accounting for continuous nature of the signal.

#### 4) time difference distribution

The final time distribution of the detected photons was obtained as the time difference $\:\left(\varDelta\:t\right)$ between the SPD time $\:\left({t}_{SPD}\right)$ and the reference detector LFS $\:\left({t}_{LFS}\right)$ as

3 $$\:\varDelta\:t=\:{t}_{SPD}-{t}_{LFS}$$

**Fig. 6:**
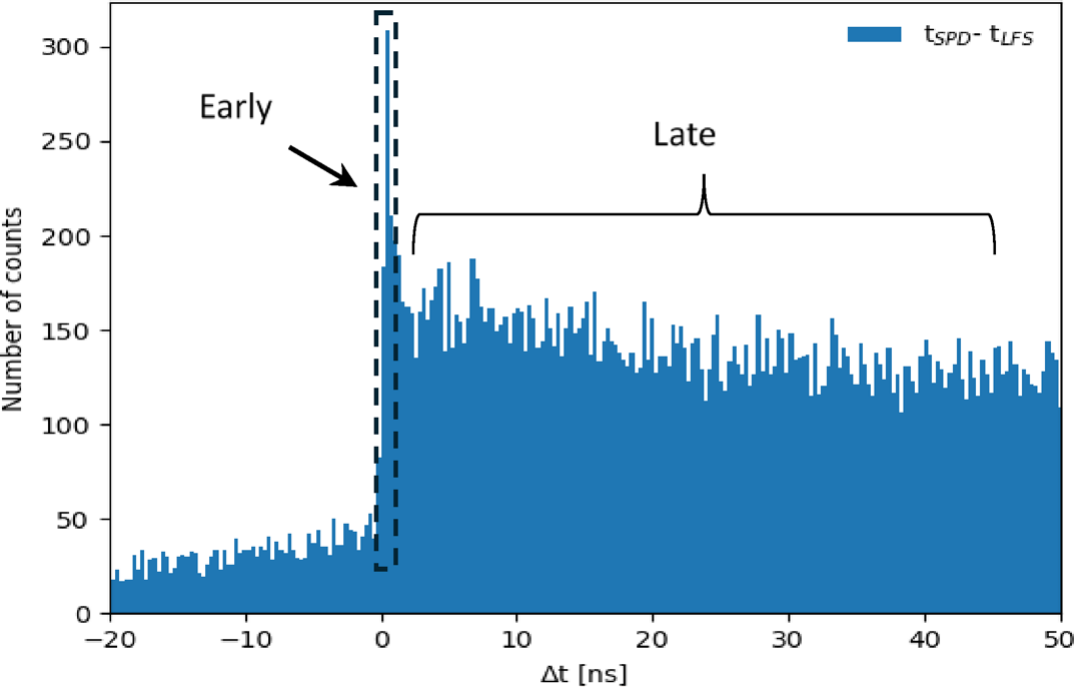
Time distribution from measurement with BGO crystal without filter shows the Cherenkov component (front peak). A fraction of slow scintillation after Cherenkov peak can be seen. Dark noise was not subtracted.

Notably, the time distribution of scintillation in the BGO crystal without filter (shown in Fig. 6) exhibited a distinct peak of prompt photons, likely associated with the Cherenkov radiation. The dark count component from the SiPM has not been subtracted from the temporal distribution. As a result, the region between − 20 and 0 ns has a non-zero component.

### Coincidence event selection

**Fig. 7:**
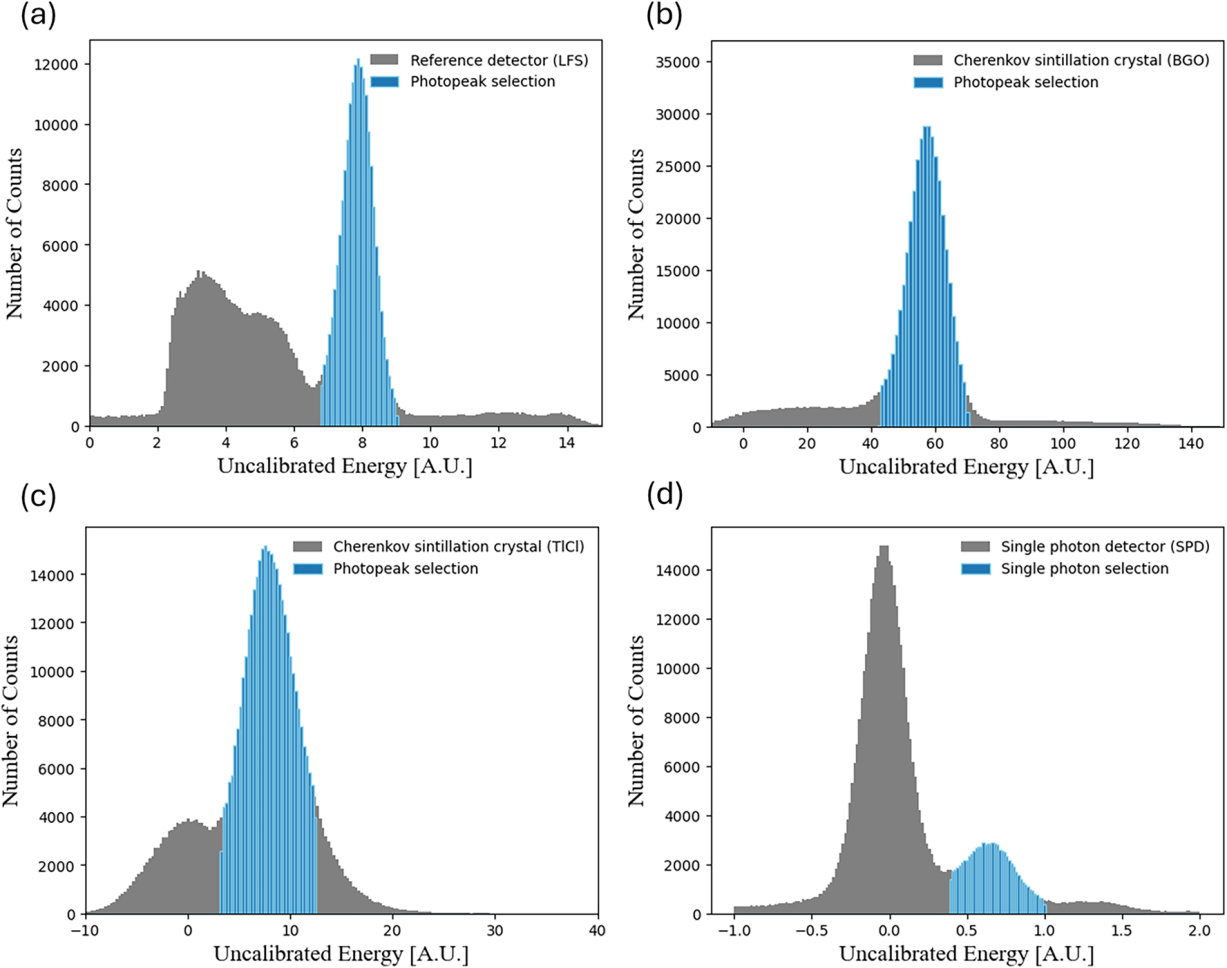
The plotted energy spectrum of the (**a**) reference detector (LFS), (**b**) Cherenkov scintillation detector (BGO), and (**c**) Cherenkov scintillation detector (TlCl) with the 511 keV photopeak selection (dark blue area) (**d**) Selected single photoelectron signal in the SPD.

The energy peak from the charge histogram was determined employing the same method as in earlier published work^23^. All events with energies less than 440/511 time the photopeak position or greater than 665/511 times the photopeak position were rejected (see Fig. 7a, b, c). Furthermore, we selected only one detected photon for SPD signals from the SiPM under test (see Fig. 7d). The charge histograms are in good agreements with the literature^9,17,38^.

### Detected photon yield

The total detected photon yield is measured to understand the possible photon loss introduced by dichroic filters. The total number of photons counts without filters, $\:{\:N}_{T}^{WOF}$, in the full-time interval (100 ns) is defined as

4 $$\:{N}_{T}^{WOF}=\left({N}_{E}^{WOF}+{N}_{L}^{WOF}\right)-{(N}_{E}^{{WOF}_{noise}}\:+{N}_{L}^{{WOF}_{noise}})$$

here, $\:{N}_{E}^{WOF}$ and $\:{N}_{L}^{WOF}$ are the early and late photon counts without filter, where the “early photon window” refers to photons arriving within 1 ns, and typically dominated by Cherenkov light, and the “late photon window” corresponding to photons arriving between 1ns and 100 ns, primarily influenced by scintillation light. $\:{N}_{E}^{{WOF}_{noise}}\:$and $\:{N}_{L}^{{WOF}_{noise}}$ correspond to the dark noise measurements without a filter for the respective early and late windows.

The overall photon count for the case with filters corresponds to the sum of early photons and late photons divided by the total number of photons without filters (Fig. 6). We compare the total number of photons with and without filters by computing the ratio:

5 $$\:\:({N}_{E}+{N}_{L}-\left({N}_{E}^{noise}+\:{N}_{L}^{noise}\right))/{N}_{T}^{WOF}$$

where $\:{N}_{E}$ and $\:{N}_{L}$ are the early and late photon counts, and $\:{N}_{E}^{noise}$, and $\:{N}_{L}^{noise}$ correspond to the different dark noise measurements with filters.

### Measurement of the Cherenkov and scintillation components

The fraction of early photoelectrons (PEs) relative to the total counts is defined as

6 $$\:{f}_{E}=\:\frac{\left({N}_{E}-{N}_{E}^{noise}\right)}{\left({N}_{t}-{N}_{t}^{noise}\right)}$$

where $\:{N}_{E}$ and $\:{N}_{E}^{noise}$ are the early PEs and the early noise PEs counts between 0 ns and 1 ns, and $\:{N}_{t}$ and $\:{N}_{t}^{noise}$ are the total PEs and the total noise PEs counts detected in the SPD within the 100 ns window.

The Cherenkov photons, $\:{N}_{C}$, are estimated as the early time component corrected by the scintillation as

7 $$\:{N}_{C}={N}_{E}-{N}_{E}^{noise}-\left[\frac{{N}_{L}-{N}_{L}^{noise}}{{T}_{L}}.{T}_{E}\right]$$

where $\:{N}_{E}$ and $\:{N}_{L}$ are the early and late photon counts. $\:{N}_{E}^{noise}$ and $\:{N}_{L}^{noise}$ correspond to the early and late noise photon counts measured by covering the SiPM with an opaque cloth, as described above. $\:{T}_{E}$ and $\:{T}_{L}$ are early and late time intervals (Fig. 6) which were chosen to be 1 ns and 99 ns, respectively. The last term in Eq. 7 accounts for the number of scintillation photons in the early window, $\:{T}_{E}$, as estimated from the late window, $\:{T}_{L}$. The selection of these time points was influenced by several factors, including experimental observation, optimizing Cherenkov photon detection, and scintillation light contribution. The $\:{T}_{E}$ and $\:{T}_{L}$ were chosen, based on the primary observed time distribution (Fig. 6) of Cherenkov and scintillation photons in our setup. The early time $\:{T}_{E}$ was chosen to be 1 ns to ensure that the detection of Cherenkov photons is maximized. The late time $\:{T}_{L}$= 99 ns was selected to encompass the majority of the scintillation light, with longer emission time compared to Cherenkov photons. Similarly, the number of scintillation photons in the full-time range, $\:{N}_{S}$, is estimated as

8 $$\:{N}_{S}=\:{N}_{L}-{N}_{L}^{noise}+\left[\frac{{N}_{L}-{N}_{L}^{noise}}{{T}_{L}}.{T}_{E}\right]$$

The scintillation contribution within the early time window ($\:{T}_{E}$) is added inside the number of scintillation photons ($\:{N}_{S}$) in Eq. (8).

The fraction of Cherenkov relative to the total photon count, $\:{f}_{C},\:$is:

9 $$\:{f}_{C}=\frac{{N}_{C}}{\left({N}_{S}\:+\:{N}_{C}\right)}$$

and the fraction of scintillation photons over the total, $\:{f}_{S}$, is

10 $$\:{f}_{S}=\frac{{N}_{S}}{({N}_{S}\:+\:{N}_{C})}$$

Finally, the ratio of Cherenkov (C) over scintillation (S) photons, R_C/S_, is

11 $$\:{R}_{C/S}=\frac{{N}_{C}}{{N}_{S}}$$

This measurement and calculations were performed for each shortpass and longpass filters and for datasets without filters.

## Results

### Transmission measurement for BGO and TlCl crystals

**Fig. 8:**
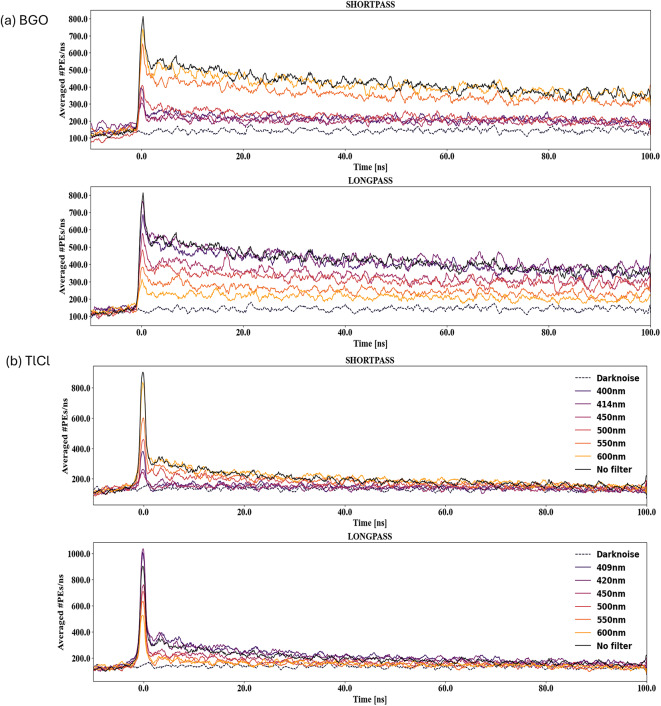
Illustrations of the averaged time distribution histogram of transmission spectra for Shortpass, and Longpass dichroic filters for (**a**) BGO (**b**) TlCl crystals. Focusing on the first 100 ns of the crystal’s scintillation kinetics where the Cherenkov signal is visible at approximately 1 ns.

The time distributions obtained for both BGO and TlCl crystals with different dichroic filters for the initial 100 ns are illustrated in Fig. 8. The noise slight increase in the early window and flattens after that (see Fig. 8). This is due to the jitter in the DRS4 trigger delays combined with the limited number of samples digitized before the trigger. A sliding window average with a 1 ns window (10 bins in 1100 bins between − 10 and 100 ns) was applied to smooth the curves. A prompt peak due to Cherenkov light detection is clearly visible in all cases. This Cherenkov light represents a small fraction of the total light production in these crystals. The time structure of signals from the BGO and TlCl crystals varied based on the type and wavelength cut-off or cut-on of the dichroic filters.

### Reflection measurement for BGO and TlCl crystals

**Fig. 9:**
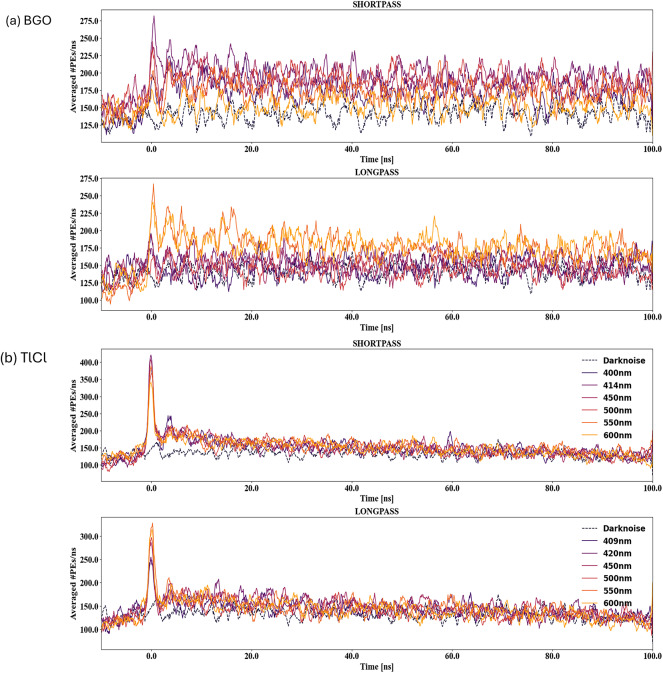
Averaged time distribution histograms of reflection spectra for shortpass and longpass dichroic filters for (**a**) BGO, (**b**) TlCl crystals. Focusing on the first 100 ns of the crystal’s scintillation kinetics, the Cherenkov signal is visible at approximately 1 ns.

The time distribution of reflection measurements was obtained for both BGO and TlCl crystals. In a similar fashion to the transmission analysis, a sliding average was used to smooth the time distributions for reflection analysis. The average time distribution histogram of the BGO crystal from reflected light, using different dichroic filters (Fig. 9a), significantly differed from the transmission time distributions histogram (Fig. 8a). This difference is due to a lower number of detected photons in the reflection configuration. The low intensity Cherenkov component for BGO crystal still provides valuable insights into precisely measuring early PEs contributions. The time distribution of reflection measurements for TlCl crystal is also presented in Fig. 9b for both shortpass and longpass dichroic filters.

### Relative light yield with and without filters

**Fig. 10:**
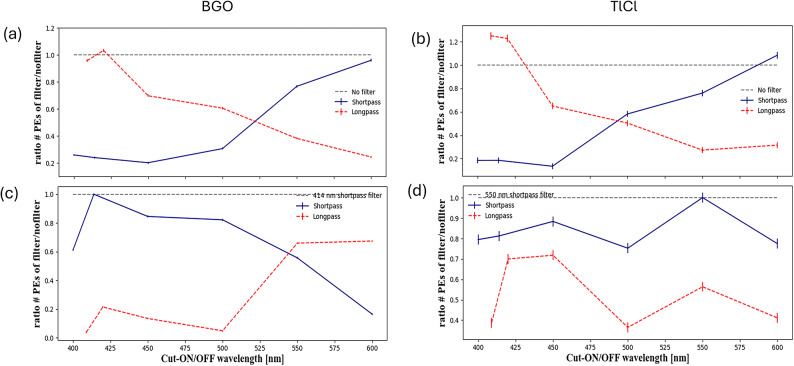
Evaluation of ratio of number of total early and late photons in the duration of 100 ns with filters over no filters. Panels (a) and (b) present for the BGO and TlCl crystals for configuration of transmission. (c) and (d) show the reflection configuration for BGO and TlCl crystal.

The ratio of the total number of detected photons with filter to those without filter is shown in Fig. 10a for BGO and Fig. 10b for TlCl for each filter in the transmission configuration. The maximum light yield was expected for the case without filter since no reflection occurred. For the reflection configuration, the light collection with filters was compared to the maximum light yield observed with a filter since reflection measurements without a filter are not possible. Shortpass filters with a cut-off wavelength of 414 nm for BGO and 550 nm for TlCl were used as references. Uncertainties were calculated assuming that the number of photons entering the SPD complies with Poisson statistics.

### Scintillation and Cherenkov ratio

**Fig. 11:**
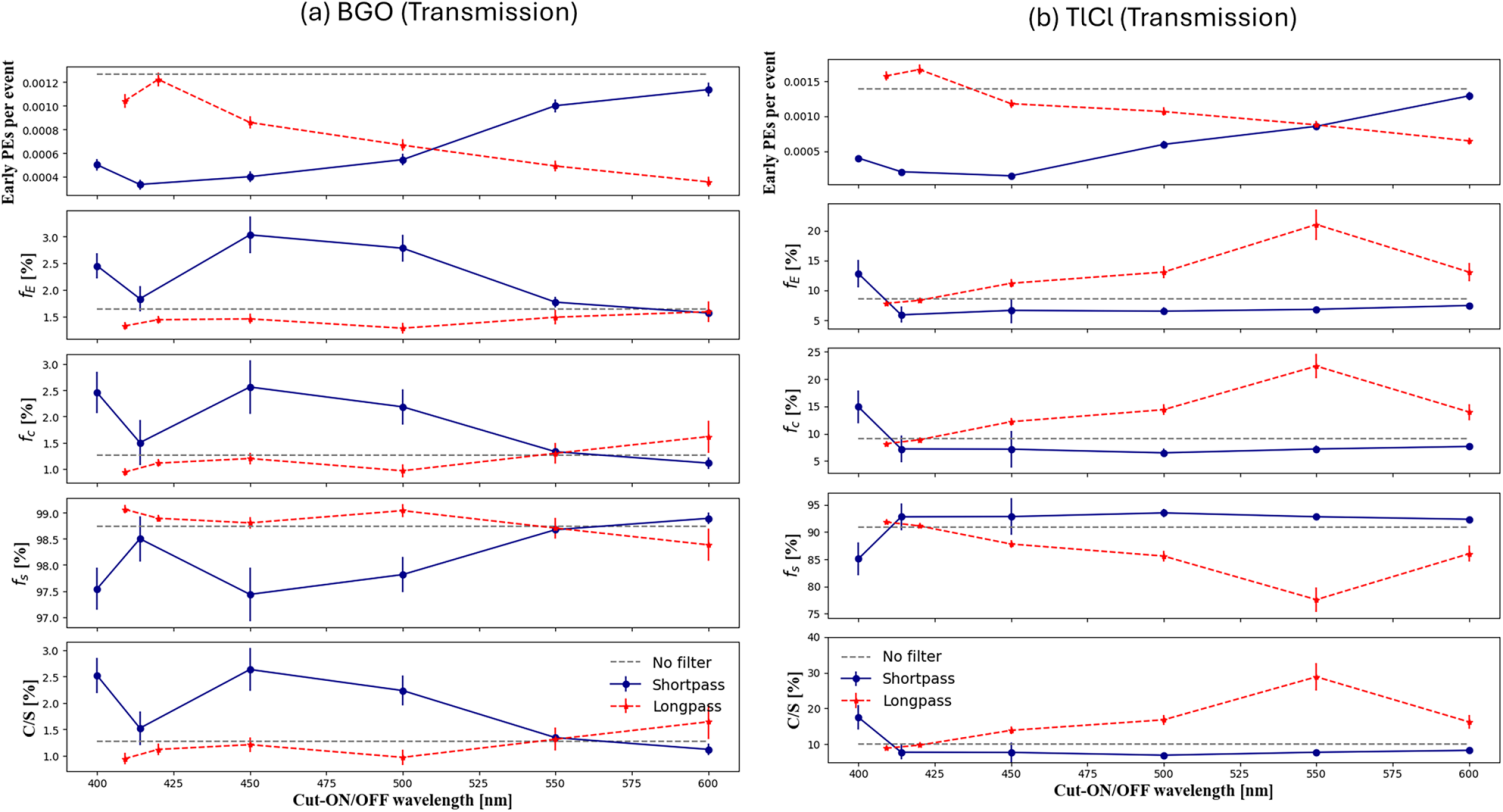
In both the longpass (dashed line) and shortpass (line) dichroic filters with transmission configurations, the number of early PEs per event in a window between 0 and 1ns, the fraction of early PEs over the total count, the fraction of Cherenkov and scintillation light and the ratio of Cherenkov photons to scintillation photons (C/S) with in the time interval 100 ns is presented for (**a**) BGO and (**b**) TlCl.

**Fig. 12:**
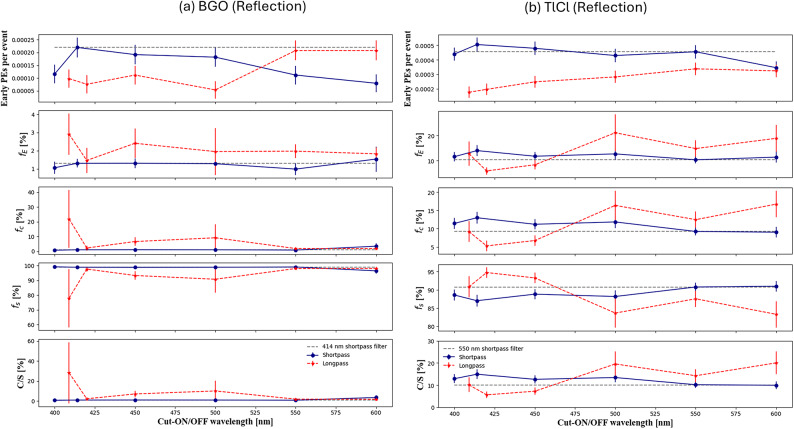
In both the longpass (dashed line) and shortpass (line) dichroic filters with reflection configurations, the number of early PEs per event in a window between 0 and 1ns, the fraction of early PEs over the total count, the fraction of Cherenkov and scintillation light and the ration of Cherenkov photons to scintillation photons (C/S) with in the time interval 100 ns is presented for (**a**) BGO and (**b**) TlCl.

For each crystal and filter, we computed the number and fraction of early photons (Eq. (6)), the fraction of Cherenkov photons (Eq. (9)), of scintillation photons (Eq. (10)), and the Cherenkov and scintillation ratios (Eq. (11)). These are shown as a function of cut-off and cut-on wavelength of dichroic filters in Figs. 11 and 12 for the transmission and reflection configurations, respectively. We could enhance the fractions of detected early photons from 1.64 ± 0.08% to 3.02 ± 0.51% for BGO with a 450 nm cut-off shortpass filter, and from 8.56 ± 0.46% to 21.01 ± 2.21% for TlCl with a 550 nm cut-on longpass filter (Fig. 11).

## Discussion

We demonstrated a method to enhance the Cherenkov over scintillation ratio in BGO and TlCl crystals using dichroic filters. For BGO, a shortpass filter with a 450 nm cut-off effectively enhances the fractions of detected early photons by a factor of approximately 1.8 in the transmission configuration (Fig. 11a) while showing an approximately 80% of the reflected photons with respect to the highest reflective filter (Fig. 10c). For TlCl, the optimal dichroic filter that provided the highest increase in early-photon ratio of 2.4 was the longpass with 550 nm cut-off for transmitted photons (Fig. 11b) while having an approximately 50% of the reflected photons with respect to the highest reflective filter (Fig. 10d).

The expected early photon detection was at 0.13 $\:{\times\:10}^{-2}$ photons per event for BGO in the transmission setup (Fig. 11a) and 0.14 $\:{\times\:10}^{-2}$ for TlCl. The maximum of the expected early PEs detected per event was for longpass filters with cut-on wavelength 409, 420 nm of (0.15 ± 0.01)$\:{\times\:10}^{-2}$and (0.16 ± 0.01) $\:{\times\:10}^{-2}$, respectively. Thereby, calculating early PEs detection per event revealed a significantly higher fraction of early photons in TlCl (21.01 ± 2.21%) compared to BGO (3.02 ± 0.51%). Since both crystals have a very similar refractive indices, the difference is due to a combination of different scintillation light yields, emission spectra, and crystal transmissivities. TlCl has a lower light yield than BGO by a factor of approximately 20, which favors Cherenkov over scintillation ratio. For BGO, the transmissivity cuts off at wavelength below 310 nm (Table 2), so the Cherenkov photons with wavelengths above 310 nm can be detected. The shortpass dichroic filters with lower cut-off wavelengths suppress (reflects) a large fraction of the scintillation, which peaks at 480 nm, while longpass filters suppress a large fraction of the Cherenkov light, as seen in Fig. 8a. Conversely, in TlCl with a cutoff wavelength of around 380 nm^17^ and a longer peak emission at 450 nm, shortpass dichroic filters suppress a large fraction of the Cherenkov light more effectively than the scintillation light (Fig. 8b), while longpass filters are more effective at enhancing the Cherenkov ratio (Fig. 8b).

In the reflection dataset, we opted for a shortpass filter as a reference rather than a mirror due to their high reflectivity and good characterization in the full range up to ~ 300 nm. Shortpass dichroic filter with a 414 nm cut-off wavelength for BGO and 550 nm for TlCl were selected as references, and the number of detected photons per event for reflection of BGO was (0.22 ± 0.04)$\:{\times\:10}^{-3}$(Fig. 12a), while the maximum expected detected photons for TlCl was for a shortpass dichroic filter with wavelength 414 nm (0.50 ± 0.0$\:5{)\times\:10}^{-3}$ compared to the reference filter (Fig. 12b). The fraction of early photons enhancement for BGO crystal was for a shortpass filter with 450 nm wavelength, however, in the case of TlCl longpass filter with wavelength of 550 nm showed the highest enhancement (Fig. 11).

The analysis in the reflection configuration shows more noise than for the transmission configuration due to the smaller effective solid angle in combination with the photosensor dark noise. The dependence of the filter properties with the reflected angle is another plausible effect that could affect light collection in the reflection configuration. In addition, the forward directionality of Cherenkov photons, which has been well documented in several studies^10,19,39,40^, versus the isotropic nature of the scintillation emission, might cause a reduction in the Cherenkov component due to geometry considerations for the reflection setup as opposed to the transmission configuration. We would like to highlight that the attenuation in dichroic filters is minimal, generally below 10%, as shown in Fig. 3 where transmissivity and reflectivity nearly add up to 100% at specific wavelengths. However, potential photon loss can occur at the interfaces between the crystal and filter, or the filter and SiPM in future TOF-PET detectors when filters are optically coupled.

We found that the total amount of detected light for TlCl for longpass filters of 409 and 420 nm is higher that the reference case without a filter (Fig. 10b). This is attributed to optical effects, such as refraction or lense distortion, that are not present without the filter.

The ratio of C/S for BGO crystal without a filter was 1.2 ± 0.1%, where we are not including the full emission time window of BGO, but only considering the first 100 ns in order to maximize the time resolution of our digitizer. The decision to limit the analysis to the first 100 ns was made to optimize the time resolution of the digitizer and to enhance the detection of early Cherenkov photons (see supplementary material for results with the full-time window). With a shortpass dichroic filter with the cut-off wavelength 450 nm in the transmission setup, the C⁄S ratio for the BGO crystal could be enhanced to 2.6 ± 0.4%. For the TlCl crystal without a filter, the C⁄S ratio was 10.02 ± 0.5%. Using a longpass dichroic filter with the cut-on wavelength of 550 nm in the transmission setup, the C/S ratio was enhanced to approximately 28.8 ± 3.7%. This enhancement implies that the C⁄S ratio for TlCl crystal exhibited an increase by a factor of approximately ~ 3. In the case of reflection for BGO crystal the ratio of C⁄S can be enhanced with a longpass dichroic filter with the cut-on wavelength 500 nm (10.09 ± 10.02%). A relatively large upward fluctuation is seen in the case of a longpass filter with the cut-on wavelength of 409 nm. This is attributed to the non-standard transmission and reflection curves for this specific filter (Fig. 3c). For the TlCl crystal in the reflection setup, the C⁄S ratio could be enhanced with longpass dichroic filters with the cut-on wavelengths of 500 nm and 600 nm to 19.5 ± 5.6% and 20.0 ± 5.1%, respectively.

In practice, dichroic filters could be implemented in a dual- readout configurations to exploit both transmitted and reflected photons. For example, complementary dichroic filters can be place at each end of a scintillator crystal, so that short or long wavelength photos are detected in each respective side. An alternative configuration is that of a dichroic filter in a 45-degree angle configuration with a downstream SiPM to collect transmitted photons, and a perpendicular SiPM to collect reflected photons. These changes to current TOF-PET configurations would be non-trivial so we leave evaluation and discussion of those for future work.

Although we have provided a proof of the principle that Cherenkov over scintillation ratio, and hence the ratio of early-photons, can be enhanced using optimized dichroic filters, the study of how this particularly quantitatively affects the time resolution in TOF-PET remains for future analyses. While dichroic filters provide a very low photon attenuation coefficient, small photon losses will still be present with respect to the case without a filter due to attenuation in the filter and interface with the crystal. This photon loss might negatively impact time resolution, and it needs to be evaluated in depth in future studies.

## Conclusion

In this work we proposed a technique to enhance the Cherenkov component over scintillation using dichroic filters in BGO and TlCl. We characterized the transmitted and reflected components and optimized the filter choice for each crystal. We successfully increased the Cherenkov over scintillation ratio by 2.17 ± 0.38 in BGO and 2.87 ± 0.40 in TlCl scintillators using a shortpass filter with the cut-off wavelength of 450 nm and a longpass filter with the cut-on wavelength of 550 nm, respectively. The fraction of early photons could be increased by a similar factor, which has potential to achieve better timing resolution in a future TOF-PET system implementing this technique. However, photon attenuation in the filters or at the interfaces between the crystal and the SiPM could negatively impact time resolution. Future studies dedicated to measure the coincidence time resolution should carefully evaluate the impact of these effects.

## Electronic supplementary material

Below is the link to the electronic supplementary material.

Supplementary Material 1

## Data Availability

All the data presented in this study are available from the corresponding author upon request. Please contact B. Mehrdel at Baharak.mehrdel@ucsf.edu for data requests.
