# Supplementary material for: Enhancing the Cherenkov over scintillation ratio using dichroic filters in BGO and TlCl for TOF-PET
Source: Sci Rep. 2025 May 28;15:18731. doi: 10.1038/s41598-025-01396-2 (PMC12120110; doi:10.1038/s41598-025-01396-2)
Supplement: Supplementary file 1 — Supplementary Material 1 [file 41598_2025_1396_MOESM1_ESM.docx]

Supplementary Materials

Enhancing the Cherenkov Over Scintillation Ratio Using Dichroic Filters in BGO and TlCl for TOF-PET

Baharak Mehrdel^1*^, Nicolaus Kratochwil^2^, Youngho Seo^1^, Jarek Glodo^3^, Pijush

Bhattacharya^3^, Gerard Ariño-Estrada^2,4^, and Javier Caravaca^5,1^

^1^Department of Radiology and Biomedical imaging, University of California San Francisco, San Francisco, CA 94107, USA

^2^Department of Biomedical Engineering, University of California at Davis, Davis, CA 95616 USA

^3^Radiation Monitoring Devices, Inc., Watertown, MA 02472, USA.

^4^Institut de Física d'Altes Energies - Barcelona Institut of Science and Technology, Bellaterra, Barcelona, Spain.

^5^Lawrence Berkeley National Laboratory, Berkeley, CA, USA

Corresponding author email: baharak.mehrdel@ucsf.edu

In this manuscript, we selected only 100 ns window to analysis the data. The decision to limit the analysis to the first 100 ns was made to optimize the time resolution of the digitizer and enhance the detection of early Cherenkov photons. This restriction is a compromise to provide sufficient pulse sampling for charge integration and energy spectrum analysis, as well as high rise time sampling for precise timing measurements.

We take into account the temporal characterization of the BGO scintillation emission in order to estimate the expected C/S ratio for a full light pulse integration. Assuming an exponential decay profile with a 300 ns decay constant, the scintillation signal would rise significantly over the extended time window. Based on this, the C/S ratio would likely decrease because the contribution of scintillation photons to the total detected light would be proportionally greater. A rough extrapolation indicates that the C/S ratio might decrease by a factor of 1.2 when integrating over the full emission time window (Fig. S1).


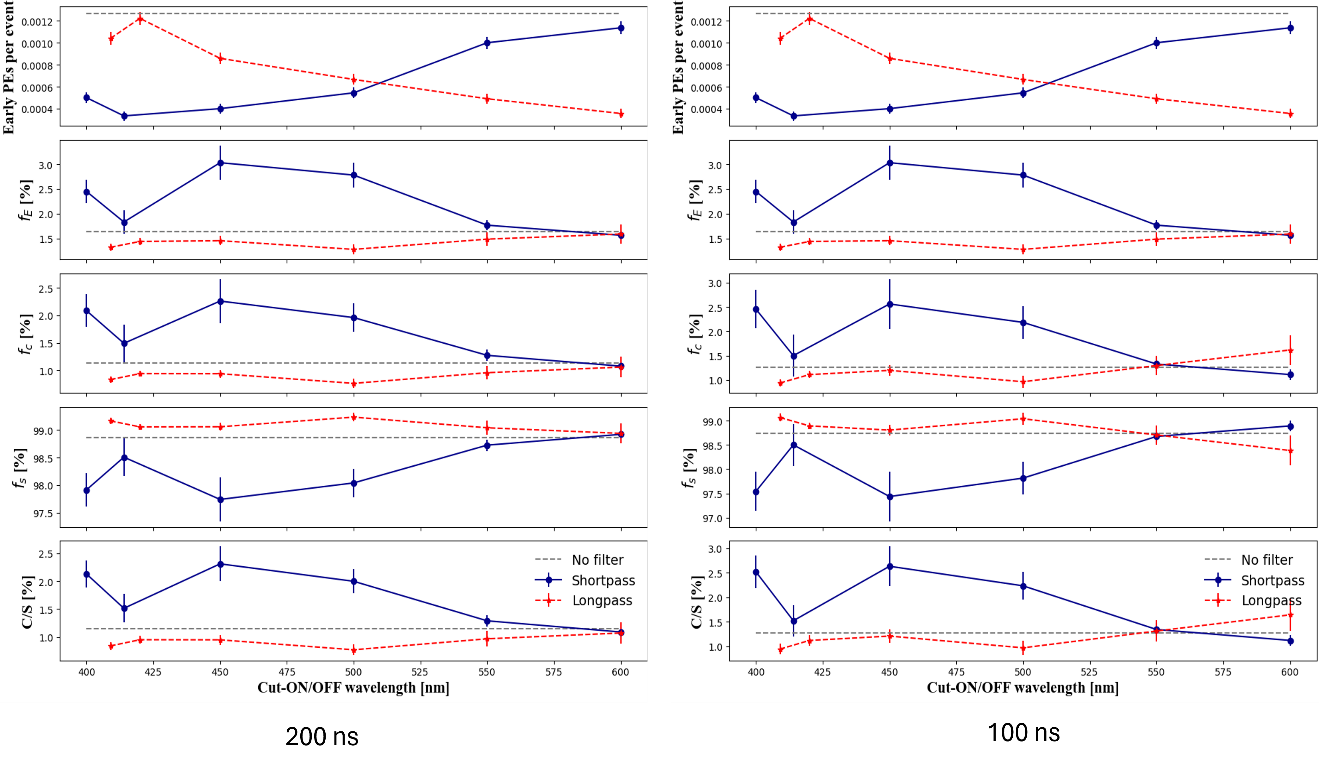

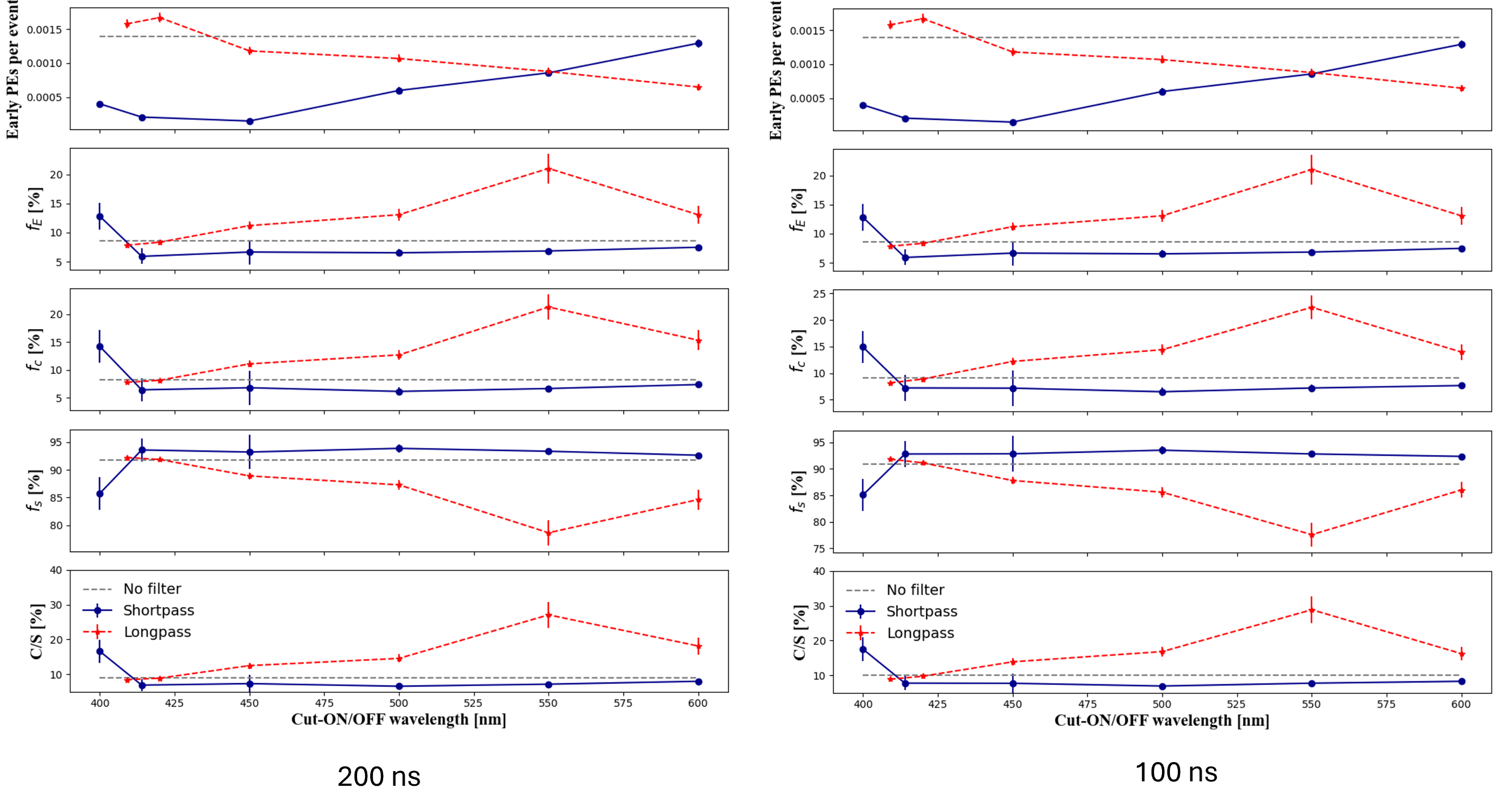


BGO - Transmission

TlCl - Transmission

(a)

(b)

Fig. S1. In both the longpass (dashed line) and shortpass (line) dichroic filters with transmission configurations, the number of early PEs per event in a window between 0 and 1ns, the fraction of early PEs over the total count, the fraction of Cherenkov and scintillation light and the ratio of Cherenkov photons to scintillation photons (C/S) with in the time interval 200 ns (left panel) and 100 ns (right panel) is presented for (a) BGO and (b) TlCl.
